# Sympathy for the Devil: Detailing the Effects of Planning-Unit Size, Thematic Resolution of Reef Classes, and Socioeconomic Costs on Spatial Priorities for Marine Conservation

**DOI:** 10.1371/journal.pone.0164869

**Published:** 2016-11-09

**Authors:** Jessica Cheok, Robert L. Pressey, Rebecca Weeks, Serge Andréfouët, James Moloney

**Affiliations:** 1 Australian Research Council Centre of Excellence for Coral Reef Studies, James Cook University, Townsville, Queensland, Australia, 4811; 2 UMR-9220 ENTROPIE, (Institut de Recherche pour le Développement, Université de la Réunion, CNRS), Laboratoire d’Excellence CORAIL, Noumea, New Caledonia; 3 College of Science and Engineering, James Cook University, Townsville, Queensland, Australia, 4811; Clemson University, UNITED STATES

## Abstract

Spatial data characteristics have the potential to influence various aspects of prioritising biodiversity areas for systematic conservation planning. There has been some exploration of the combined effects of size of planning units and level of classification of physical environments on the pattern and extent of priority areas. However, these data characteristics have yet to be explicitly investigated in terms of their interaction with different socioeconomic cost data during the spatial prioritisation process. We quantify the individual and interacting effects of three factors—planning-unit size, thematic resolution of reef classes, and spatial variability of socioeconomic costs—on spatial priorities for marine conservation, in typical marine planning exercises that use reef classification maps as a proxy for biodiversity. We assess these factors by creating 20 unique prioritisation scenarios involving combinations of different levels of each factor. Because output data from these scenarios are analogous to ecological data, we applied ecological statistics to determine spatial similarities between reserve designs. All three factors influenced prioritisations to different extents, with cost variability having the largest influence, followed by planning-unit size and thematic resolution of reef classes. The effect of thematic resolution on spatial design depended on the variability of cost data used. In terms of incidental representation of conservation objectives derived from finer-resolution data, scenarios prioritised with uniform cost outperformed those prioritised with variable cost. Following our analyses, we make recommendations to help maximise the spatial and cost efficiency and potential effectiveness of future marine conservation plans in similar planning scenarios. We recommend that planners: employ the smallest planning-unit size practical; invest in data at the highest possible resolution; and, when planning across regional extents with the intention of incidentally representing fine-resolution features, prioritise the whole region with uniform costs rather than using coarse-resolution data on variable costs.

## Introduction

Systematic conservation planning (hereafter “conservation planning”) can be described in stages from stakeholder engagement through to the application and maintenance of conservation actions [1, 2]. A key stage in conservation planning is prioritising important areas for biodiversity conservation (hereafter “prioritisation”). Conservation prioritisations are important in guiding efficient investment of limited resources to design protected areas and off-park interventions for conservation [3, 4]. Prioritising allows planners to quantitatively assess the importance of sites for conservation action, while also explicitly considering aspects of their socioeconomic context. Prioritisations are typically based on data on biodiversity and, more recently, socioeconomic costs, coupled with predefined quantitative objectives for environmental classes, species, or processes of interest [5, 6]. By “environmental classes” we refer to spatial subdivisions of terrestrial, freshwater, or marine environments, based on physical, climatic, and/or biological variables, with the aim of deriving environmental surrogates (sensu [1]) for conservation planning. The planning region is then subdivided into planning units, subdivisions of planning regions that are assessed and compared to identify priorities. Data on biodiversity and socioeconomic costs, intersected with planning units, and combined with objectives, are analysed by decision-support tools that determine low- or least-cost conservation designs [7].

Almost half of the stages involved in conservation planning require decisions about spatial scale [8], with most related to prioritisation. Prioritisation inherently involves analysing spatial data [4], which can be collected and analysed at multiple resolutions (vagueness of the term ‘scale’ may confuse discussing its effects, so we define related terms in Table 1). A number of studies, mostly terrestrial, have explored the influence of planning-unit size and thematic resolution of environmental classes on prioritisation (e.g., [9–12], and see [13–15] for marine studies). While there is a growing body of evidence for the influence of aspects of resolution on prioritisation outputs, studies so far have mainly focused on the individual effects of spatial resolution of data, thematic resolution of environmental classes, and size of planning units, with few examining the effects of combinations of these factors. Importantly, no studies have yet considered how socioeconomic cost data can also interact with all these other factors to influence the selection of prioritised areas. With our increasing recognition of the importance of considering socioeconomic costs in prioritisations [3, 16–18], and the potential for conservation designs to guide conservation actions, it is necessary to investigate the potential interactions that can occur between planning-unit size, thematic resolution of environmental classes, and variability of cost data.

**Table 1:** Definitions of key terms used related to scale in conservation planning.

Key term	Definition
**Spatial extent**	The total area covered by an exercise in analysis or planning, or the planning domain.
**Resolution** [19]	The precision used in measurement, or the interval between observations or categories, in either space or time.
**Spatial resolution**	The minimum spatial unit of measured information.
**Thematic resolution** [20]	The number of themes or categories per unit area. Thematic resolution can range from very coarse (few distinct classes) to very fine (many distinct classes). When applied to environmental classifications, thematic resolution is the number of environmental classes within a planning domain.
**Fine-resolution prioritisation**	Typically associated with prioritisations across small spatial extents, ranging from 10^−3^ to 10^1^ km^2^ [21], and using relatively small planning units and data at high spatial resolution, and if used, categorical data at high thematic resolutions.
**Coarse-resolution prioritisation**	Typically associated with prioritisations across large spatial extents, ranging from 10^3^ or 10^4^ km^2^ upwards [21], and using relatively large planning units and data at low spatial resolution, and if used, categorical data at low thematic resolutions.

Another question related to spatial scale remains unexplored: the degree to which conservation priorities defined at fine resolutions are likely to be nested within those defined at coarse resolutions. Within the last two decades, an increasing number of studies have undertaken prioritisations at global or regional extents, using entire ecoregions as planning units [5, 22–28], or very large planning units (e.g., 772 km^2^ squares [29], 900 km^2^ squares [30]). A critical limitation of planning across such large spatial extents is that, due to resource limitations and practicality, it is generally not feasible to collect, map or analyse biodiversity data at very fine spatial and thematic resolutions [12]. Therefore, prioritisations conducted across large spatial extents typically employ both coarse-resolution data and large planning units (“coarse prioritisations”, Table 1). Despite the shortcomings associated with prioritising in this way [9, 11, 12, 14], coarse prioritisations continue to be produced because of the desire for broad views of conservation priorities [6, 31–34].

As a strategy to overcome mismatches between coarse priorities and the much finer resolutions at which conservation actions often need to be applied (see [8]), coarse prioritisations have been conceived as a starting point to guide subsequent finer-resolution assessments [35, 36]. This approach assumes, usually implicitly, spatial nestedness: that priority areas identified with large planning units and low thematic resolutions will necessarily contain priority areas that would be identified using small planning units and high thematic resolutions. This assumption implies that closer investigations of extensively-defined priorities will identify all fine-resolution priorities. The two studies, to our knowledge, that have investigated this assumption have found that coarse assessments can represent many finer-resolution priorities, except in heterogeneous or fragmented areas [9, 12]. However, key gaps apparent from these studies are that the findings were not consistent (fine-resolution priorities were represented to varying extents), and data were for terrestrial environments only. Moreover, neither of these studies considered the role of socioeconomic cost data, and how these could influence the nestedness of fine-resolution priorities within coarse ones.

With so many aspects of data interacting in the prioritisation process across multiple extents or resolutions, the devil is in the detail. Important details that remain to be explored are the potential interactions between three prioritisation factors: 1) size of planning units, 2) thematic resolution of environmental classes, and 3) spatial variability in socioeconomic costs, and how these together influence prioritisation outputs, particularly in terms of the spatial configuration of priorities when different cost layers are used. These interactions likely have important implications for identifying effective prioritisation strategies. Here, we investigate the interacting effects of these three factors on conservation prioritisations in marine planning contexts, where environmental classes are used commonly as proxies for biodiversity. Specifically, we use geomorphological reef classes (hereafter “reef classes”). We assess the relative effect of each of these factors (research objective 1), and, for the first time to our knowledge, interactions occurring between all three (research objective 2), on (i) the total extent and cost of reserve solutions, and (ii) spatial configurations of priority areas. We also assess the ability of coarse prioritisations to adequately represent finer-resolution priorities (research objective 3), in terms of (i) the spatial nestedness of priorities determined at different resolutions, and (ii) the extent of incidental representation of reef classes at high thematic resolution by coarse-resolution priorities. Using case studies from marine environments provides insights to support the rapidly increasing number of marine protected areas in response to targets under the Convention on Biological Diversity [37]. In doing so, we help to understand the devil in the detail of marine conservation prioritisation.

## Methods

### Study regions

**Fig 1:**
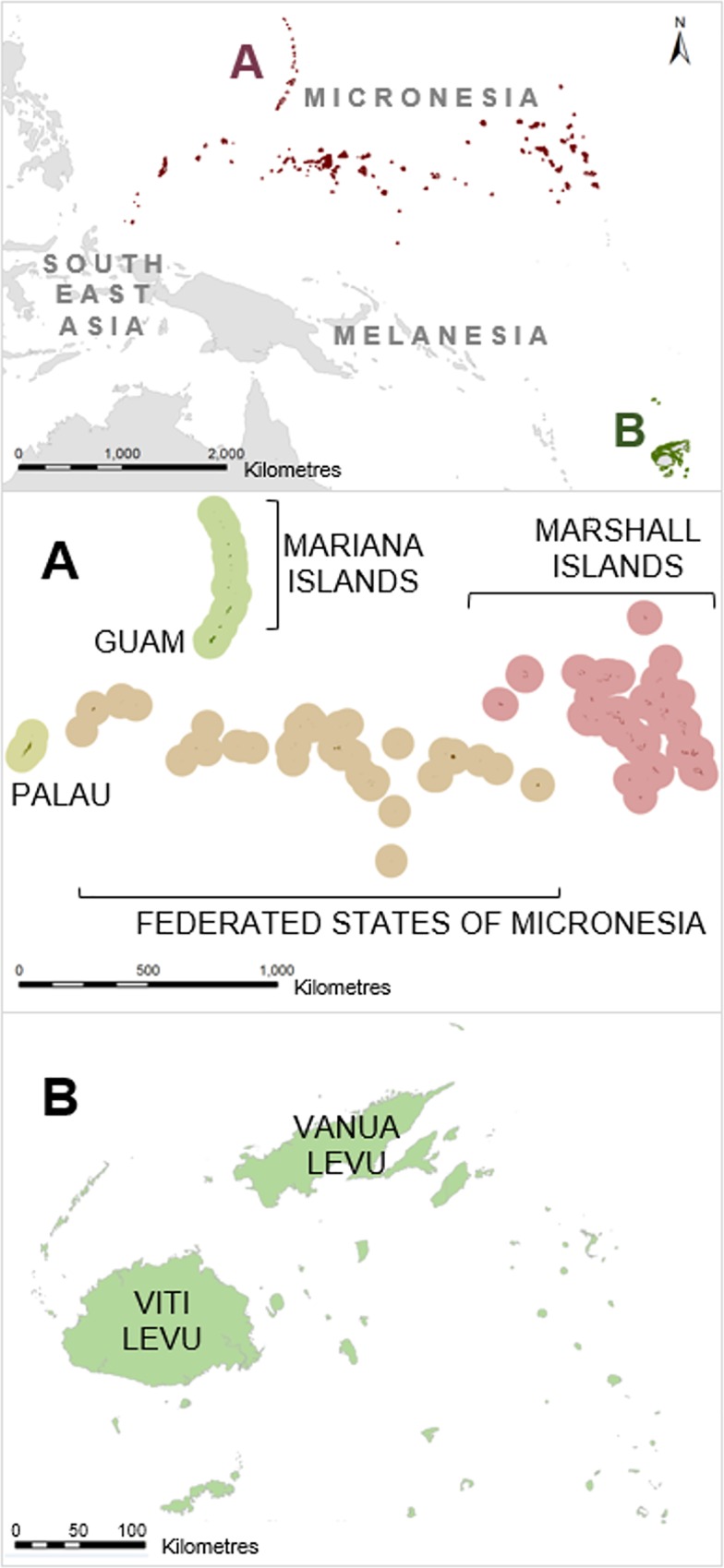
**Regional context and enlarged maps of the two study regions: (A) Micronesia and (B) Fiji.** Buffers are shown around Micronesian nations to increase visibility of the numerous small coral islands and atolls.

Two regions were used as case studies: Fiji and Micronesia (consisting of the Mariana Islands, Marshall Islands, Palau, Guam, and the Federated States of Micronesia [FSM]; Fig 1). The individual Micronesian nations were considered as one region for the purposes of our analyses to provide a large, regional extent. The total extent of the planning regions for Fiji and Micronesia were approximately 24,439 km^2^ and 32,168 km^2^, respectively. The focus of this study was on all coral reefs contained within each country’s exclusive economic zone. Aspects of planning-unit size and thematic resolution of reef classes (hereafter “thematic resolution”) are especially relevant in archipelagic and developing nations, where there is a pressing need for conservation action but the mismatch between regional-level planning and local-level implementation is large [38]. This disparity between regional and local perspectives is primarily attributed to the often coarsely-defined prioritisations [39] and the often fine (devolved) spatial resolutions of governance in these developing regions, with complex social, economic, and political factors shaping conservation decisions (see [8, 13, 38, 40]).

Though our analyses are grounded in real data, they are demonstration exercises, not intended to inform real-world conservation action in our study regions. For this reason, we did not consider existing marine protected areas in Fiji or Micronesia. The benefits of using empirical rather than modelled data (in the case of the maps of reef classes) are that the results from this study will be a more realistic representation of outcomes expected in real-world applications.

### Study design

**Fig 2:**
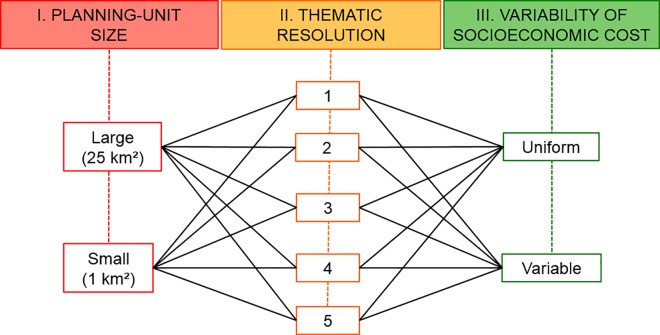
Study design showing tested factors, factor levels, and the 20 unique combinations between all levels.

We examined three prioritisation factors: planning-unit size (analysed at two levels), thematic resolution (five levels), and spatial variability of socioeconomic costs (two levels). A full factorial design (giving a total of 20 unique prioritisation scenarios; Fig 2) was employed to determine the influence of each of these factors in combination with the others. We used a scenario coding system (Table 2) to facilitate interpretation of subsequent results.

**Table 2:** Coding system to identify individual scenarios. Codes are assigned to each level of each prioritisation factor. An example scenario code based on this coding system is “L1U”: large planning units (L), first level of thematic resolution (1), and uniform cost (U).

	Prioritisation-factor level	Level code
**Planning-unit size**	Large	L
	Small	S
**Thematic resolution**	1 (coarse)	1
	2	2
	3	3
	4	4
	5 (fine)	5
**Variability of socioeconomic cost**	Uniform	U
	Variable	V

### Planning-unit size

**Fig 3:**
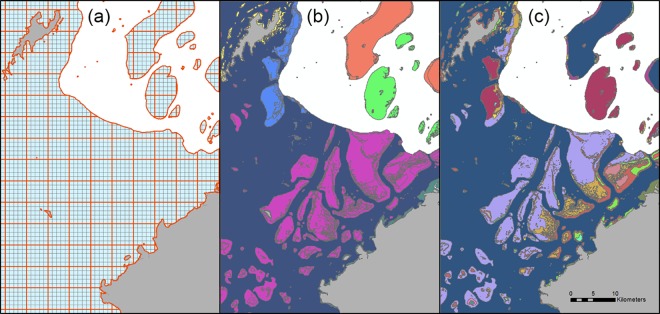
Example maps of planning-unit sizes and thematic resolutions explored in the Fiji dataset. All maps represent the same spatial extent and location; grey polygons represent Fiji terrestrial areas (islands). (a) Planning-unit sizes: ‘small’ (1 km^2^; blue squares) and ‘large’ (25 km^2^; red squares); 25 small planning units are nested within each large, non-edge planning unit. Note that both planning-unit grids were clipped to all reef areas, resulting in irregular planning units on the perimeters. (b) & (c) Examples of two of the five levels of thematic resolution: (b) level 2 (11 total reef classes in Fiji), and (c) level 4 (43 total reef classes in Fiji).

Two planning-unit sizes were explored: 1 km^2^ (‘small’) and 25 km^2^ (‘large’). These totalled 29,781 small and 1,845 large planning units for Fiji, and 38,019 small and 2,339 large planning units for Micronesia. Square planning units were used so that the smaller planning units spatially nested within the larger ones (Fig 3A). The sizes were determined by a review of marine prioritisation exercises to realistically gauge ‘small’ and ‘large’ planning-unit sizes, relative to real-world contexts. Planning units were complete squares, except where they occurred on the edges of reefs. Edge planning units were trimmed to reef edges so that the area of each planning unit was equal to the extent of the reef it contained. This was important for calculating uniform cost from reef area for each planning unit (see section “Socioeconomic cost data” below).

### Thematic resolution

Reef classes of Fiji and Micronesia were mapped using high spatial resolution Landsat 7 ETM+ satellite images (30 metres) as part of the Millennium Coral Reef Mapping Project [41]. However, a mapped minimum discernible unit is much larger, around 10,000 m^2^ (or a cluster of about 10 pixels). The reef classes were mapped separately for each Micronesian country, with some reef classes in common between countries. For our analyses, we considered each reef class from each country as unique, regardless of nominal thematic overlap with the other Micronesian countries. In other words, an atoll in Palau is considered here as a different reef class than an atoll in Marshall Islands or FSM. Practically, this means that prioritised areas were forced to be spread between all the different Micronesian countries. Doing so was necessary to realistically reflect the individual objectives that the separate countries would have in such transnational-scale planning exercises, while still providing a large regional extent for the prioritisation scenarios. Distinguishing reef classes between countries was also precautionary for the use of reef classes as biodiversity surrogates. Differences in species associated with the same reef class in different countries are likely to arise from dissimilar reef complexities between the different Micronesia regions, and the distance decay of similarity in ecological communities [42].

The maps describe reef geomorphology in a hierarchical classification scheme with five thematic resolutions, from levels 1 to 5, with level 1 referring to the lowest resolution (2 reef classes in Fiji; 4 reef classes in Micronesia), and level 5 to the highest resolution (280 reef classes in Fiji; 181 reef classes in Micronesia). The 4 and 181 reef classes for level 1 and level 5, respectively, in Micronesia reflect subdivision of reef classes by country. Example maps are in Fig 3B and 3C.

### Socioeconomic cost data

**Fig 4:**
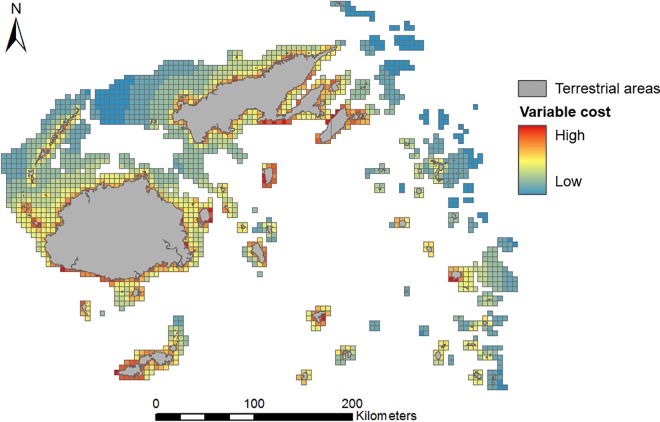
Example map showing distribution of cost variability across the Fiji planning region. Values, shown here for large planning units, are based on distance to fisher populations as a proxy for opportunity cost.

Our two layers of socioeconomic opportunity cost [18] were: spatially uniform, with planning-unit cost proportional to area (i.e., amount of reef contained within); or spatially variable, modelled to represent a proxy of opportunity cost to fishers (Fig 4). Since opportunity cost data did not exist for the whole of either study region, we created spatially variable cost layers for both regions using weighted linear distance from fisher populations (derived from census information; [43–48]) to the furthest reef areas (details in S1 File). Distance measures have commonly been used as a proxy for socioeconomic cost in previous studies [18, 49], although we acknowledge that they are limited predictors of actual opportunity costs to fishers [49]. However, our aim here was to contrast two different cost layers, not to guide conservation planning for implementation.

### Priority-setting tool parameters and calibrations

Across all scenarios, the conservation objective was to protect 30% of each reef class (both Fiji and Micronesia have made commitments to protect 30% of their inshore waters [50, 51]). To identify sets of planning units that achieved this objective, we used the decision-support software Marxan [52]. Marxan uses a simulated annealing algorithm with iterative improvement to find spatial reserve designs that meet biodiversity objectives for the least socioeconomic cost.

For each scenario, we ran Marxan to produce 100 solutions (i.e., giving us 100 replicates). With 20 scenarios, this produced a total of 2000 individual solutions for comparison. Marxan also produces a ‘selection frequency’ output, which records the number of times each planning unit was selected across multiple solutions. Planning units with higher selection frequency are more likely to be needed to achieve a set of conservation objectives. The “Species Penalty Factors” in Marxan were calibrated for each scenario so that all solutions achieved the overall objective of 30% of each reef class (minimum proportion met > 0.999), following the Marxan Good Practices Handbook [53].

### Output comparison and statistical analyses

Multiple output comparisons and analyses were performed to achieve each of our research objectives (Table 3). All statistical analyses were performed using the statistical software package, *R* [54]. To compare configurations of solutions from scenarios with different planning-unit sizes, each large planning unit was converted to its component small planning units (maximum 25). In the case of selection frequencies, the smaller parts of large planning units were given the same values as their larger planning unit; so if a large planning unit had a selection frequency of 50, each of the (up to) 25 smaller planning units nested within it also had a value of 50. For comparison of individual solution outputs, if a large planning unit was selected, all of the smaller planning units nested within it were also considered selected.

**Table 3:** Summary of output comparisons and statistical analyses for each research objective.

Research objective	Output comparison / statistical analyses
(1) Assess relative effect of each factor	• Total reserve extent and cost of solutions• Spatial configuration of priority areas
(2) Assess interactions between factors	
(3) Assess the ability of coarse prioritisations to represent finer-resolution priorities	• Spatial nestedness of priorities at different resolutions
	• Extent of incidental representation of fine thematic-resolution objectives by coarse-resolution priorities

#### Research objectives 1 & 2: total reserve extent and cost of solutions

There are two fundamental metrics from Marxan reserve solutions: the overall extent of the reserve solution and total cost of the selected areas. These metrics reflect the overall spatial and cost efficiency of the reserve solutions. We compared these two metrics between all scenarios. Costs were compared as proportions of maximum possible cost (of the whole planning region) to allow direct comparisons of scenarios using different cost layers.

#### Research objectives 1 & 2: spatial configuration of priority areas

**Fig 5:**
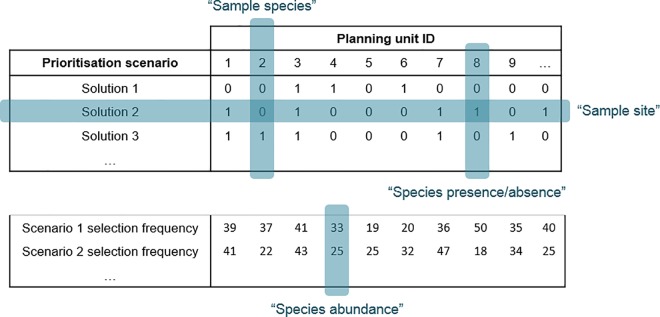
Example of Marxan output data. For calculation of dissimilarity, we regarded Marxan individual solutions as analogous to biological sampling sites and planning units as analogous to recorded species. For single Marxan solutions, planning units were either selected (“present”) or unselected (“absent”). For selection frequencies across 100 replicate solutions in a scenario, entries for planning units were equivalent to species abundance data.

Output data from Marxan are analogous to ecological data (Fig 5). Therefore, all individual scenario solutions (2000 in total, 100 per scenario) were compiled into a data matrix for statistical analyses with prioritisation scenarios or “sites” as rows and planning units or “species” as columns. Another data matrix was created for all selection frequency outputs (20 in total, one per scenario). Another analogy between Marxan output data and community data is that the matrix contains many zero entries for “species” (or planning units). The data matrices were therefore Hellinger-transformed, which allowed meaningful use of parametric ordination methods (which are Euclidean-based), while circumventing the problems associated with Euclidean distance to analyse matrices with many zeros (see [55] for details). We used parametric ordination methods because of the higher level of statistical power possible with such tests.

Once the data matrices were transformed, a Euclidean dissimilarity matrix was calculated (‘vegan’ *R* package; [56]). This involved measuring, pair-wise, the dissimilarities (or distance) between all 2000 solutions (presence-absence) or all 20 solutions (selection frequencies). From these dissimilarity matrices, we used an average-linkage hierarchical cluster analysis to determine, through visual interpretation, the spatial similarity between solutions and whether any of the tested factors (i.e., planning-unit size, thematic resolution, and variability of costs) appeared to influence similarity (‘sparcl’ *R* package for plotting; [57]). The cluster analysis on the selection frequency outputs allowed us to check whether the clusters derived from individual solutions were affected by spatially idiosyncratic solutions.

The dissimilarity matrix was also used to conduct a redundancy analysis (RDA), or constrained ordination (‘vegan’ *R* package), to quantify the extent to which the tested factors explained the clusters, since this information is absent from the cluster analysis itself. We used an RDA because it focuses only on the variation that can be explained by the ‘environmental’ variables (in this case, our prioritisation factors: planning-unit size, thematic resolution, and cost variability). To test whether the results from the ordination model were statistically significant, we ran a permutation test on the RDA (‘vegan’ *R* package).

#### Research objective 3: spatial nestedness of priorities

**Fig 6:**
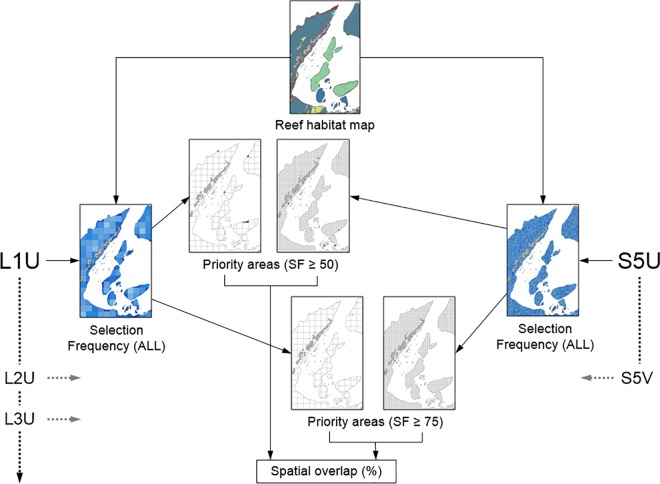
The flow of analyses related to spatial nestedness. Analyses described for spatial nestedness, defined here as extent of overlap of high-priority areas based on large planning units (left) with small planning units with high thematic resolution and both cost layers (right). Dotted arrow lines indicate repetition of the same analyses across all other coarse-scenarios performed with both of the test scenarios, S5U and S5V.

We determined the degree to which fine-resolution priorities (small planning units, high thematic resolution) were spatially nested within all coarse-resolution priorities (large planning units), some of which were based on mapping at coarse thematic resolutions. We achieved this by comparing priorities produced from each scenario with large planning units (10 scenarios in total) with two of the finest-resolution prioritisation scenarios: S5V (small planning units, thematic resolution level 5, variable costs) and S5U (small planning units, thematic resolution level 5, uniform costs) (Fig 6). Since the research objective was to ascertain the ability of coarse-resolution priorities produced with large planning units to spatially capture fine-resolution priorities produced with small planning units, we examined the two prioritisation factors of potential influence: thematic resolution and cost variability. Our scenarios with large planning units were intended to reflect, to varying degrees, the coarse prioritisations usually necessary over large extents. Our two “test” scenarios with small planning units were intended to reflect the higher-quality data usually available only after closer study of relatively localised parts of large planning regions (except that our high-quality data covered whole planning regions in this instance).

To assess the extent of spatial nestedness, we examined the amount of spatial overlap between high-priority areas defined at two levels: planning units with selection frequencies of ≥ 50 and ≥ 75 (Fig 6). Extent of spatial nestedness was indicated by the percentage of small high-priority planning units from each of the two test scenarios that overlapped with high-priority large planning units from the ten scenarios being tested.

#### Research objective 3: incidental representation of fine thematic-resolution objectives by coarse-resolution priorities

To complement the analyses of nestedness, we wanted to know the degree to which the conservation objective (i.e., 30% of each reef class) for the highest thematic resolution (level 5) would be incidentally achieved by priority areas identified by the ten scenarios using large planning units. The planning-unit selection frequencies for all scenarios using large planning units were converted to probabilities of being selected (see Table 4 for example, method modified from [58]). When summed across all planning units, this method gave the expected areas of each level 5 reef class that would be represented by scenarios based on large planning units. Then we plotted the relationship between incidental representation of level 5 reef classes and their rarity in our study regions, with rarity determined by the extent of each reef class relative to that of the whole study area, expressed as a percentage: [1 –(total reef class extent / total planning extent)] × 100. This formula gave large percentages to very restricted reef classes and smaller values to more extensive reef classes.

**Table 4:** Method calculating the expected areas of level 5 reef classes that would be selected for reservation in each of the scenarios using large planning units.

Planning unit ID (PUID)	Selection frequency	Probability of selection (*P*)	Level 5 reef class code	Reef class area (*A*) (km^2^)	Expected area of level 5 code selected (P*A) (km^2^) by PUID
1	45	0.45	1	0.395	A1,1 = 0.178
1	45	0.45	5	0.375	A1,5 = 0.169
1	45	0.45	7	0.230	A1,7 = 0.104
2	33	0.33	2	0.012	A2,2 = 0.004
2	33	0.33	7	0.988	A2,7 = 0.326
3	21	0.21	7	0.132	A3,7 = 0.028
3	21	0.21	9	0.868	A3,9 = 0.182
…					

## Results

All analyses indicated similar influences and interactions of factors for both regions (Micronesia and Fiji). Therefore, only results for the Fiji case study are presented here. Results for the Micronesia case study are presented in S1–S5 Figs.

### Individual effects of prioritisation factors

#### Total reserve extent and cost of solutions

**Fig 7:**
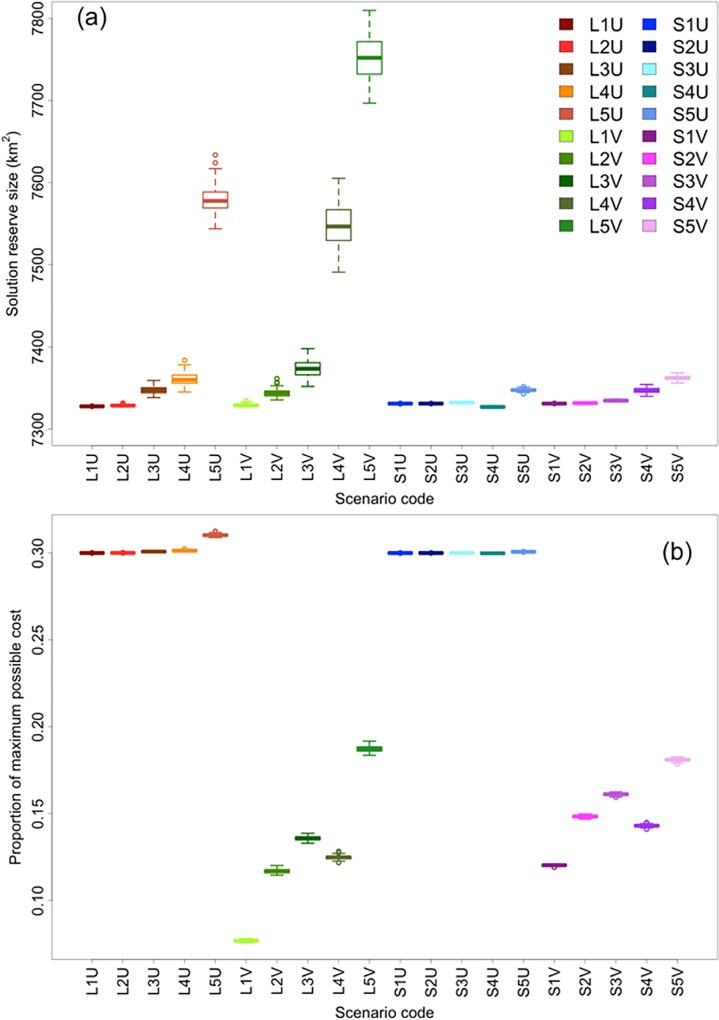
Comparisons of total reserve size and proportions of maximum possible cost. (a) Boxplots of ranges of reserve solution sizes for each scenario based on 100 replicate runs. (b) Boxplots of ranges of total costs (expressed as proportions of maximum possible cost) for each scenario based on 100 replicate runs. Each change in shade of the same colour represents the change in thematic resolution (always presented in order from level 1–5, left to right) for each combination of planning-unit size and cost variability. Colour scheme representing all scenarios remains the same throughout all figures to facilitate interpretation.

We found a general trend of increasing total selected reserve extent with increasing thematic resolution, regardless of planning-unit size or variability of cost (Fig 7A). However, there was an obvious difference in the rate of increase in total reserve size between the different planning-unit sizes as thematic resolution increased. The rate of increase was markedly higher with larger planning units.

The total costs of solutions, expressed as proportion of maximum possible cost (both uniform and variable), generally increased with thematic resolution (Fig 7B). Overall, total reserve costs were substantially lower in all scenarios with variable cost. For scenarios with variable cost, the only exception to the general increasing trend in cost with increasing thematic resolution was at level 4, for which total reserve cost decreased (i.e., scenarios L4V and S4V). This trend was less apparent in the Micronesia results (S1 Fig).

#### Spatial configuration of priority areas

**Fig 8:**
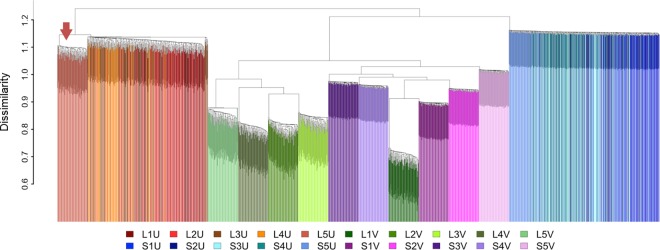
Spatial dissimilarity between all 2000 solutions for the Fiji case study.

The 2000 individual solutions were clustered mainly by cost variability and planning-unit sizes (Fig 8), confirming that these factors were important in determining spatial configurations. Distinct clusters first formed (lowest dissimilarity) between scenarios with variable cost, forming separate groups of clusters primarily between large and small planning units (green and purple clusters, Fig 8). Variability of cost data appeared to have the largest effect on spatial dissimilarity between solutions, based on the dissimilarity distances and the distinct difference in clustering of solutions with and without variable cost.

**Fig 9:**
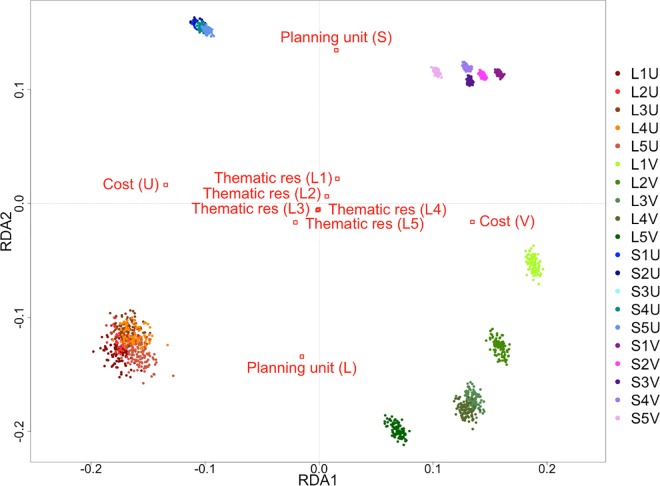
Comparison of spatial variation between all solutions produced using RDA for the Fiji case study. Cost variability mainly explains variation along RDA1, while variation along RDA2 is mostly represented by different planning-unit sizes. Red squares are centroids of the different prioritisation-factor levels, representing the average amount of spatial variance that lines up with the plotted axes.

The two RDA axes explained 87.71% of the spatial variance due to the three prioritisation factors tested (Fig 9). Axis RDA1 represented most of the variation due to variability in cost, evidenced by the wide horizontal separation between scenarios prioritised with uniform costs (left) and variable costs (right). A marked difference between scenarios with uniform cost and those with variable cost is the clear separation of variable-cost scenarios according to thematic resolution (Fig 9), reflecting the clustering in the hierarchical analysis. Axis RDA2 mainly represented the variation due to planning-unit size, with large planning-unit scenarios in the lower part of the plot and small planning-unit scenarios in the upper part (Fig 9). Another observable difference between large and small planning-unit sizes is the amount of spatial dissimilarity between solutions for the same scenarios. Larger planning units (lower) produced individual solutions for combinations of cost variability and thematic resolution that were more loosely clustered, and therefore less similar in configuration, than the same combinations with small planning units (upper). There was no clear association between the spatial variance caused by thematic resolution with either of the plotted axes. The plotted centroids (red squares; Fig 9) of each prioritisation-factor level in the biplot indicate the average amount of spatial variance, relative to both axes, predicted for all solutions of the same prioritisation-factor level. The locations of the centroids for each thematic-resolution level indicate the relatively small overall influence of thematic resolution on spatial differentiation between solutions (Fig 9). The modelled influences of the three factors on spatial dissimilarity of all solutions from the ordination analysis were statistically significant (Table 5).

**Table 5:** Results of the permutation test showing significance of each of the tested prioritisation factors in influencing the spatial dissimilarity of solutions.

	df	Variance	*F*-statistic	p-value
Cost variability	1	0.0742	260.879	<0.001
Planning-unit size	1	0.0267	93.815	<0.001
Thematic resolution	4	0.0149	13.126	<0.001

### Interaction effects between prioritisation factors

#### Total reserve extent and cost of solutions

The increase in reserve size with increasing thematic resolution appeared to be amplified for scenarios involving variable cost (Fig 7A). Similarly, scenarios with variable cost involved notable increases in total cost with increasing thematic resolution, while there was relatively little effect of planning-unit size or thematic resolution on the total cost of reserves for scenarios with uniform cost (Fig 7B).

#### Spatial configuration of priority areas

In the clusters of variable-cost scenarios formed in the hierarchical cluster analysis (Fig 8), differentiation between the different levels of thematic resolution was clear, evidenced by the largely separate clusters forming between the shades of green and purple. In other words, all the solutions for a given combination of variable cost and thematic resolution were clustered together, with very little or no overlap with the other variable-cost scenarios. The next major cluster contained solutions with uniform cost and small planning units (blue cluster, Fig 8). Here, there was much less spatial differentiation between solutions for different thematic resolutions, evidenced by the mixing of blue shades within the cluster. The last major cluster to form and the most spatially distinct from all other solutions, contained solutions with uniform cost and large planning units (red cluster, Fig 8). Similar to the scenarios with uniform cost and small planning units, there was no spatial differentiation between thematic resolutions with the exception of level 5 (red arrow; Fig 8). In summary, the distinctiveness of solutions for different thematic resolutions depended on whether uniform or variable costs were used.

Within the broad trend of spatial differentiation produced between solutions with uniform cost (left) and variable cost (right) in the RDA analysis, distinct clusters formed from solutions based on variable costs, composed of different levels of thematic resolution (green and purple points; Fig 9), as observed in the cluster analysis (Fig 8). Also akin to the cluster analysis, solutions produced by uniform-cost scenarios, to the left of the biplot, were not distinguished by thematic resolution (red and blue points). This is apparent from the grouping of solutions with small (blue) and large (red) planning units, regardless of thematic resolution.

### Ability of coarse prioritisations to represent finer-resolution priorities

#### Spatial nestedness of S5U priorities

**Fig 10:**
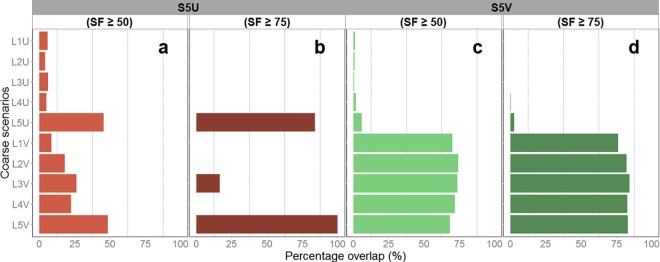
Nestedness of high-priority small planning units (test scenarios) within high-priority areas defined by large planning units. Nestedness of S5U high-priority areas, defined at: (a) selection frequency ≥ 50, and (b) selection frequency ≥ 75. Nestedness of S5V high-priority areas, defined at: (c) selection frequency ≥ 50, and (d) and selection frequency ≥ 75.

Spatial nestedness of S5U selected areas (calculated as percentage of S5U high-priority areas within large high-priority planning units) appeared to be influenced mainly by matched thematic resolution (Fig 10A and 10B). Nestedness of S5U areas was highest within large planning units selected to represent level 5 reef classes, for both selection frequency ≥ 50 (45–48%; Fig 10A) and selection frequency ≥ 75 (84–100%; Fig 10B).

#### Spatial nestedness of S5V priorities

Spatial nestedness of S5V selected areas (calculated as above) was strongly influenced by variable cost (Fig 10C and 10D). Nestedness within coarse scenarios that used variable cost was high: 68–74% (high priority = selection frequency ≥ 50; Fig 10C) and 76–84% (high priority = selection frequency ≥ 75; Fig 10D). Nestedness within coarse scenarios that used uniform cost was much lower: 0.7–6% (high priority = selection frequency ≥ 50) and 0–3% (high priority = selection frequency ≥ 75). Unlike the spatial nestedness of S5U priorities, thematic resolution did not appear to influence nestedness of S5V priorities.

#### Incidental representation of fine thematic-resolution objectives by coarse planning-unit scenarios with uniform cost

**Fig 11:**
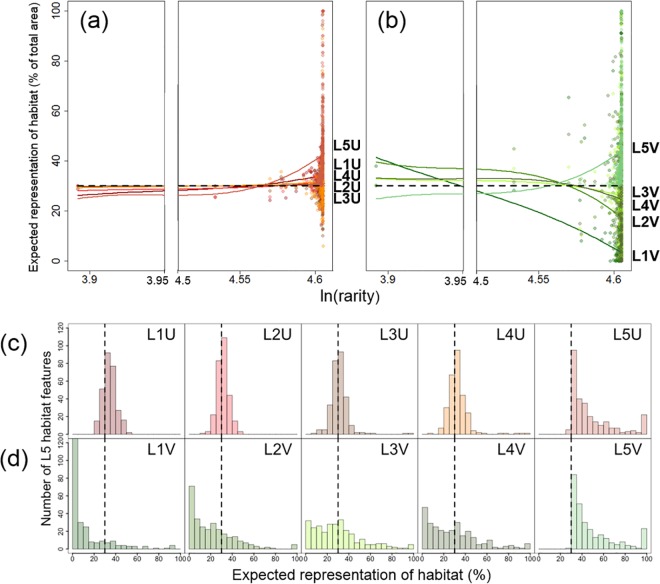
Incidental representation of level 5 reef classes by scenarios using large planning units. (a-b) Scatter plots showing expected representation of each level 5 reef class (as a percentage of total area of reef class occurrence) for each coarse scenario with (a) uniform cost and (b) variable cost, in relation to reef class rarity (transformed to natural log). Due to spread and left-skewness of rarity values, plots are shown with *x*-axis breaks where no data occur to facilitate interpretation. Local regression (LOESS) curves were fitted for each coarse scenario, indicating non-linear trends in each scatter plot. Dashed horizontal lines represent the 30% objective for level 5 reef classes. (c-d) Histograms showing the distributions of expected representation of level 5 classes for coarse scenarios with (c) uniform cost and (d) variable cost, plotted with 5% bin widths. Dashed vertical lines represent the 30% objective for level 5 classes.

Uniform-cost scenarios based on thematic resolution levels 1–4 had incidental representation of level 5 reef classes that trended close to the 30% objective across rarity values (Fig 11A), with variation above and below that value for individual reef classes (and see Fig 11C). For the scenario based on level 5, over-achievement of the 30% objective increased with reef class rarity (Fig 11A), reaching as high as 100% for some very rare reef classes, with virtually no under-achievement (Fig 11C).

#### Incidental representation of fine thematic-resolution objectives by coarse planning-unit scenarios with variable cost

Variable-cost scenarios based on thematic resolution levels 1–4 achieved more variable incidental representation of level 5 reef classes and much lower achievement of the overall objective than scenarios with uniform cost (Fig 11B and 11D). The level 1–4 scenarios with variable cost produced much lower values for rarer reef classes, frequently achieving no incidental representation, and there was more variation around the fitted curves than those based on uniform cost (Fig 11B). For the coarse scenario based on level 5 reef classes, there was the same increase in over-achievement of objectives as for uniform cost (Fig 11B), also reaching 100% at high rarity, and the same lack of underachievement (Fig 11D).

## Discussion

We sought to understand the detail underlying the influence of three factors–planning-unit size, thematic resolution of reef classes, and spatial variability of cost data–on the outputs from conservation prioritisation. Our findings have implications for future marine prioritisations, particularly those that use extensive, coarse-resolution assessments as guides for conservation actions at finer resolutions.

### Individual effects of prioritisation factors

Variability of cost data had the largest influence on planning outputs. Scenarios produced with variable cost data produced solutions that were less costly relative to all scenarios with uniform cost (in terms of the calculated proportions of maximum possible cost). This result accords with those of previous studies (e.g., [14, 49, 59]). Spatially variable costs underlie a consistent spatial bias of selection toward cheaper planning units, where there are choices to achieve objectives.

Cost variability also had the strongest influence on the spatial configuration of solutions. It is known that the costs used in a decision-support tool such as Marxan have a large influence on the selection of planning units, and that different cost data can lead to dissimilar patterns of priorities [60]. Of the studies that have explicitly compared the spatial differences in priorities determined with different socioeconomic cost layers, all observed an influence of cost [49, 61–65]. Our analyses further demonstrate that variable cost data had a greater overall influence on configuration of priority areas than planning-unit size or thematic resolution. This result reaffirms the importance of selecting appropriate cost metric(s) for identifying conservation priorities.

Larger planning units substantially increased total reserve extent required to meet objectives. Pressey and Logan (1998) [11] interpreted this result as the larger above-objective representation of environmental classes with larger planning units because of reduced precision in sampling parts of classes. Rouget (2003) [12] and Hamel et al. (2013) [13] also found that large planning units were less efficient in terms of area needed in meeting conservation objectives. Spatial dissimilarity between all solutions was also considerably influenced by planning-unit size. This is not completely unexpected considering the potential disparities in total reserve extent attributable to planning-unit size. Other studies support this finding with the observation that changes in planning-unit size yield spatially different priority areas [66–68]. Different planning-unit sizes producing diverging priority areas are likely due to the spatial inflexibility, relative to features being represented, that occurs when planning-unit size increases [66], reflected in the commonly observed accompanying loss of spatial efficiency [68, 69].

Thematic resolution of reef classes had the least overall influence on priorities. The only consistent direct effect of this factor was the increase in total reserve extent with increase in resolution, regardless of planning-unit size or cost variability. This occurs because of the lack of fit between planning units and the boundaries of environmental classes [10], which is exacerbated when larger planning-unit sizes and finer, more detailed thematic resolutions are used. Despite having the least direct influence on priorities, the most significant influence of thematic resolution occurred in combination with other prioritisation factors examined in this study, highlighting the importance of exploring these interactions.

### Interaction effects between prioritisation factors

The most notable interaction between the three factors we examined was between cost variability and thematic resolution. We found that solutions with variable cost (which were less costly overall) were more sensitive to increasing thematic resolutions than those with uniform cost. This result reflects the profiles of variable cost within reef classes. At lower thematic resolutions, there are more spatial options for achieving objectives and a higher probability that objectives can be achieved with relatively cheap planning units. At higher thematic resolutions, spatial options for achieving objectives are more constrained, and more expensive planning units are required to achieve objectives for at least some of the rarer reef classes that result from thematic subdivision. However, it is important to note that the exception to this trend was with thematic resolution level 4, which actually resulted in a decrease in total cost compared to level 3, despite having a greater number of reef classes. This is due to the nature of reef classifications used for level 4, which were not uniquely hierarchical within level 3 classes (in other words, the same level 4 classes existed under different level 3 categorical classes). Thus the level 4 reef classes are more widespread over the planning domain, leaving more options to find lower cost solutions. We know of one previous study that has also demonstrated an interaction between thematic resolution and the spatial variability of cost in terms of cost efficiency [70].

The change in interaction effect found when increasing thematic resolution from level 3 to level 4 demonstrates the relevance of the relationship between the spatial distribution of environmental classes and cost values, and how this can influence the ability to achieve cost efficiency in reserve solutions. This is further supported by the fact that level 4 had a higher number of rare reef classes compared to level 3, which, all other data aspects being equal, should result in increased total reserve cost [71]. The consistent decrease in total cost seen with level 4 compared to level 3, when prioritised with variable cost only, suggests that two other factors are in play. First, the cost profiles of the rare reef classes in level 4 could be lower than those in level 3 (i.e., rare classes in level 4 can be found within less costly planning units), leaving more scope to achieve objectives in lower-cost planning units. Second, there could be higher spatial co-occurrence of rare reef classes in level 4, which would essentially increase the spatial (and therefore cost) efficiency of meeting the objectives for these rare classes in fewer planning units. A weaker signal of reduced total variable cost for level 4 reef classes was apparent in the Micronesia results, demonstrating the region-specific nature of interactions between prioritisation factors influencing planning outputs.

Another aspect of the interaction between thematic resolution and variable cost was their combined influence on spatial differentiation between priorities. The mechanism behind changes in spatially variable costs determining different spatial priorities [7, 61] makes this finding understandable. A spatially variable cost layer constrains the choices of planning units (to cheaper ones, where possible) to meet the increasing number of reef class objectives with increasing thematic resolution. This constraint leads to spatially distinct sets of planning units among thematic resolutions because, at each thematic resolution, the spatial relationship between cost and reef classes is different. It should be noted again that this is likely to arise only if planning-unit options for certain classes are constrained due to the spatial pattern of expensive planning units in the cost layer. Conversely, with uniform cost, selection of planning units is more spatially flexible, leading to less distinct sets of planning units between thematic resolutions.

The other notable interaction occurred between planning-unit size and thematic resolution. Large planning units interacted with increasing thematic resolution to significantly increase total reserve extent required to achieve the overall 30% objective, particularly towards the higher resolutions. This interaction effect was considerably less evident in the scenarios with small planning units. Again, this is likely due to the greater spatial mismatch that occurs between the larger planning units and the boundaries of the finer-resolution environmental classes [10, 12], resulting in less spatial precision and flexibility in achieving the same objectives.

### Ability of coarse prioritisations to represent finer-resolution priorities

Our results indicate that coarse prioritisations are unreliable guides to fine-resolution priorities, unless the same socioeconomic data are used at both resolutions. This presents an inherent risk, since planning at regional scales using variable cost data will almost certainly involve coarse-resolution surrogates for cost [72] that are unlikely to reflect variables important to people on the ground [8, 14, 38, 70]. Put another way, nestedness of priorities would be expected only if fine-resolution priorities used the same limited, coarse-resolution surrogates for cost applied across whole regions, and this would require planners to ignore local insights into actual costs. Updating cost data on the ground as coarse-resolution priorities are investigated would inevitably change the cost data on which those coarse priorities were based [21]. While allowing local fine-tuning of priority areas, this would also undermine the very basis for the coarse priorities.

Given that achievement of conservation objectives is the basic aim of systematic conservation planning, incidental representation of finer-resolution environmental classes by coarse prioritisations is at least as important as the spatial nestedness of priorities. Our analysis on the extent of incidental representation of level 5 reef classes demonstrated that all scenarios prioritised with uniform cost outperformed scenarios with variable cost. Whilst some previous studies similarly found reasonable potential for coarse prioritisations to achieve fine-resolution objectives [9, 12], these did not consider variable socioeconomic cost data in their prioritisations. Bridge et al. (2016) [73], however, found a significant negative relationship between planning-unit cost and incidental representation of environmental classes in the Great Barrier Reef marine reserve network, Australia. The less spatially-biased selection of planning units that occurs when no (or uniform) costs are considered allows a greater chance for incidental representation of finer-resolution objectives, with this effect being almost indistinguishable between the different levels of thematic resolution tested in this study.

With our increasing understanding of the ability of cost data to influence conservation priorities [61, 74, 75], we must recognise the imperative to use accurate and representative cost layers in conservation prioritisations. Such data rarely exist across extensive regions, due to the expense and complicated logistics involved in obtaining them. It is therefore critical that we formulate strategies to help circumvent this data limitation, based on our understanding of the relative influence of prioritisation factors and the potential interactions that can occur between them. Based on our findings, one such strategy could be to prioritise with spatially uniform costs when planning across large regional extents and then update priorities as finer-resolution data on biodiversity and costs become available, as coarse-resolution planning transitions to fine-resolution implementation [8]. Our results indicate that using uniform costs for initial regional-scale planning in an adaptive planning process will increase the incidental representation of finer-resolution objectives, through the initial selection of planning units that are not biased towards planning units that appear cheaper with coarse, and likely inaccurate, regional-scale surrogates.

Importantly, we found that confidence in incidental representation was lower for fine-resolution reef classes that were less extensive in our study areas. Similar findings have come from previous studies [9, 58, 73, 76]. As rare environmental classes or species tend to be at most risk of destruction or extinction [77, 78], this is a critical point to consider regarding the ability of coarse prioritisations to represent fine priorities.

## Conclusions

With increasing calls for conservation planning to incorporate socioeconomic costs [3, 14, 16, 17], our findings lead to three recommendations. First, where regular planning units are employed, the smallest practical planning-unit size will maximise spatial, and therefore, cost efficiency. Second, wherever possible, planners should invest in accurate biodiversity and socioeconomic cost data (or surrogates) at the highest resolution possible. While these two recommendations come generally at the price of practicality or expense of data acquisition, our third recommendation considers situations where the first two are not feasible. When planning across regional extents and when incidental representation of fine-resolution (or unmapped) environmental classes is desirable, it is better to prioritise the whole region with uniform costs if subsequent finer prioritisations will follow. Otherwise, spatially variable cost data can bias selection of planning units enough to reduce the likelihood of incidental representation of fine-resolution environmental classes. The importance of incorporating socioeconomic cost data is now commonly touted in the conservation planning literature. However, we have found that the cost data used in conservation prioritisation can be so influential on the configurations of selected areas, that failing to recognise appropriate scales at which to incorporate cost data can lead to negative consequences for biodiversity.

While we have shown that the influence of planning-unit size, thematic resolution, and cost variability have generally consistent impacts on conservation prioritisation outputs, interactions between these factors can lead to surprising results. Although consistent findings from both our study regions, involving very different reef complexities, suggests the findings might be generalised to other situations, it should be noted that the reef-class mapping for the two regions originates from the same mapping project and uses the same method [41]. Our study adds to the growing body of evidence on these interactions, but repeated studies of this nature with reef-class data that are categorically non-hierarchical and unrelated, along with different types of socioeconomic cost data, would be valuable in better understanding the potential interactions that can occur. Further understanding the details on how these problems propagate throughout the prioritisation process is relevant to achieving more effective and efficient conservation solutions in the face of expanding loss of global biodiversity and natural environments, and waning conservation resources.

## Supporting Information

S1 Fig Comparisons of total reserve size and proportions of maximum possible cost. (a) Boxplots of ranges of reserve solution sizes for each scenario based on 100 replicate runs. (b) Boxplots of ranges of total costs (expressed as proportions of maximum possible cost) for each scenario based on 100 replicate runs. Each change in shade of the same colour represents the change in thematic resolution (always presented in order from level 1–5, left to right) for each combination of planning-unit size and cost variability. Colour scheme representing all scenarios remains the same throughout all figures to facilitate interpretation.
(TIF)

S2 Fig Spatial dissimilarity between all 2000 solutions for the Micronesia case study. (TIF)

S3 Fig Comparison of spatial variation between all solutions produced using RDA for the Micronesia case study. Planning-unit size mainly explains variation along RDA1, while variation along RDA2 is mostly represented by cost variability. Red squares are centroids of the different levels of tested factors, representing the average amount of spatial variance that lines up with the plotted axes.
(TIF)

S4 Fig Nestedness of high-priority small planning units (test scenarios) within high-priority areas defined by large planning units. Nestedness of S5U high-priority areas, defined at: (a) selection frequency ≥ 50, and (b) selection frequency ≥ 75. Nestedness of S5V high-priority areas, defined at: (c) selection frequency ≥ 50, and (d) selection frequency ≥ 75.
(TIF)

S5 Fig Incidental representation of level 5 reef classes by scenarios using large planning units. (a-b) Scatter plots showing expected representation of each level 5 reef class (as a percentage of total area of feature occurrence) for each coarse scenario with (a) uniform cost and (b) variable cost, in relation to reef class rarity (transformed to natural log). Due to spread and left-skewness of rarity values, plots are shown with *x*-axis breaks where no data occur to facilitate interpretation. Local regression (LOESS) curves were fitted for each coarse scenario, indicating non-linear trends in each scatter plot. Dashed horizontal lines represent the 30% objective for level 5 reef classes. (c-d) Histograms showing the distributions of expected representation of level 5 classes for coarse scenarios with (c) uniform cost and (d) variable cost, plotted with 5% bin widths. Dashed vertical lines represent the 30% objective for level 5 classes.
(TIF)

S1 File Additional methods for creating the spatially variable socioeconomic cost layer for Fiji and Micronesia. (DOCX)

## References

[1] Margules CR, Pressey RL. Systematic conservation planning. Nature. 2000;405(6783):243–53. 10.1038/35012251 10821285

[2] Pressey RL, Bottrill MC. Approaches to landscape- and seascape-scale conservation planning: convergence, contrasts and challenges. Oryx. 2009;43(4):464–75.

[3] Carwardine J, Wilson KA, Watts M, Etter A, Klein CJ, Possingham HP. Avoiding costly conservation mistakes: the importance of defining actions and costs in spatial priority setting. PLoS One. 2008;3(7):e2586 10.1371/journal.pone.0002586 18596914 PMC2440517

[4] Margules CR, Pressey RL, Williams PH. Representing biodiversity: data and procedures for identifying priority areas for conservation. J Biosci. 2002;4(2):309–26. 10.1007/BF02704962 12177531

[5] Green A, Smith SE, Lipsett-Moore G, Groves C, Peterson N, Sheppard S, et al Designing a resilient network of marine protected areas for Kimbe Bay, Papua New Guinea. Oryx. 2009;43(4):488–98.

[6] Rouget M, Cowling RM, Lombard AT, Knight AT, Kerley GIH. Designing large-scale conservation corridors for pattern and process. Conserv Biol. 2006;20(2):529–61. 10.1111/j.1523-1739.2006.00297.x 16903115

[7] Watson JEM, Grantham HS, Wilson KA, Possingham HP. Systematic conservation planning: past, present and future In: Ladle RJ, Whittaker RJ, editors. Conservation Biogeography: Wiley-Blackwell; 2011 p. 136–60.

[8] Mills M, Pressey RL, Weeks R, Foale S, Ban NC. A mismatch of scales: challenges in planning for implementation of marine protected areas in the Coral Triangle. Conserv Lett. 2010;3(5):291–303.

[9] Payet K, Rouget M, Lagabrielle E, Esler KJ. Measuring the effectiveness of regional conservation assessments at representing biodiversity surrogates at a local scale: a case study in Réunion Island (Indian Ocean). Austral Ecol. 2010;35(2):121–33.

[10] Pressey RL, Logan VS. Reserve coverage and requirements in relation to partitioning and generalisation of land classes: analyses for Western New South Wales. Conserv Biol. 1995;9(6):1506–17.

[11] Pressey RL, Logan VS. Size of selection units for future reserves and its influence on actual vs targeted representation of features: a case study in western New South Wales. Biol Conserv. 1998;85(3):305–19.

[12] Rouget M. Measuring conservation value at fine and broad scales: implications for a diverse and fragmented region, the Agulhas Plain. Biol Conserv. 2003;112(1):217–32.

[13] Hamel MA, Andréfouët S, Pressey RL. Compromises between international habitat conservation guidelines and small-scale fisheries in Pacific island countries. Conserv Lett. 2013;6(1):46–57.

[14] Richardson EA, Kaiser MJ, Edward-Jones G, Possingham HP. Sensitivity of marine-reserve design to the spatial resolution of socioeconomic data Conserv Biol. 2006;20(4):1191–202. 16922235 10.1111/j.1523-1739.2006.00426.x

[15] Van Wynsberge S, Andréfouët S, Gaertner-Mazouni N, Remoissenet G. Conservation and resource management in small tropical islands: trade-offs between planning unit size, data redundancy and data loss. Ocean Coast Manag. 2015;116:37–43.

[16] Ban NC, Hansen GJA, Jones M, Vincent ACJ. Systematic marine conservation planning in data-poor regions: socioeconomic data is essential. Mar Policy. 2009a;33(5):794–800.

[17] Ban NC, Mills M, Tam J, Hicks CC, Klain S, Stoeckl N, et al A social-ecological approach to conservation planning: embedding social considerations. Front Ecol Environ. 2013;11(4):194–202.

[18] Naidoo R, Balmford A, Ferraro PJ, Polasky S, Ricketts TH, Rouget M. Integrating economic costs into conservation planning. Trends Ecol Evol. 2006;21(12):681–7. 10.1016/j.tree.2006.10.003 17050033

[19] Scholes RJ, Reyers B, Biggs R, Spierenburg MJ, Duriappah A. Multi-scale and cross-scale assessments of social-ecological systems and their ecosystem services. Curr Opin Environ Sustain. 2013;5(1):16–25.

[20] Dalleau M, Andréfouët S, Wabnitz CCC, Payri C, Wantiez L, Pichon M, et al Use of habitats as surrogates of biodiversity for efficient coral reef conservation planning in Pacific Ocean Islands. Conserv Biol. 2010;24(2):541–52. 10.1111/j.1523-1739.2009.01394.x 20105207

[21] Pressey RL, Mills M, Weeks R, Day JC. The plan of the day: managing the dynamic transition from regional conservation designs to local conservation actions. Biol Conserv. 2013;166:155–69.

[22] Beger M, McGowan J, Heron SF, Treml EA, Green A, White AT, et al Identifying conservation priority gaps in the Coral Triangle Marine Protected Area System Brisbane, Australia 57pp: Coral Triangle Support Program of USAID, The Nature Conservancy, and The University of Queensland, 2013.

[23] Jenkins CN, Joppa L. Expansion of the global terrestrial protected area system. Biol Conserv. 2009;142(10):2166–74.

[24] Klein CJ, Ban NC, Halpern BS, Beger M, Game ET, Grantham HS, et al Prioritising land and sea conservation investments to protect coral reefs. PLoS One. 2010;5(8):e12431 10.1371/journal.pone.0012431 20814570 PMC2930002

[25] Miclat EFB, Ingles JA, Dumaup JNB. Planning across boundaries for the conservation of the Sulu-Sulawesi Marine Ecoregion. Ocean Coast Manag. 2006;49(9):597–609.

[26] Olson DM, Dinerstein E. The global 200: priority ecoregions for global conservation. Ann Mo Bot Gard. 2002;89(2):199–224.

[27] Olson DM, Dinerstein E, Wikramanayake ED, Burgess ND, Powell GVN, Underwood EC, et al Terrestrial ecoregions of the world: a new map of life on Earth. Bioscience. 2001;51(11):933–8.

[28] Spalding MD, Fox HE, Allen GR, Davidson N, Ferdaña ZA, Finlayson M, et al Marine ecoregions of the world: a bioregionalisation of coastal and shelf areas. Bioscience. 2007;57(7):573–83.

[29] Alpine JE, Hobday AJ. Area requirements and pelagic protected areas: is size an impediment to implementation? Mar Freshw Res. 2007;58(6):558–69.

[30] Venter O, Fuller RA, Segan DB, Carwardine J, Brooks T, Butchart SHM, et al Targeting global protected area expansion for imperiled biodiversity. PLoS One. 2014;12(6):e1001891. 10.1371/journal.pbio.1001891 PMC4068989 24960185

[31] Beger M, McGowan J, Treml EA, Green AL, White AT, Wolff NH, et al Integrating regional conservation priorities for multiple objectives into national policy. Nat Commun. 2015;6(8208). 10.1038/ncomms9208 PMC4579602 26364769

[32] Klein C, Wilson K, Watts M, Stein J, Berry S, Carwardine J, et al Incorporating ecological and evolutionary processes into continental-scale conservation planning. Ecol Appl. 2009;19(1):206–17. 19323184 10.1890/07-1684.1

[33] Poiani KA, Richter BD, Anderson MG, Richter HE. Biodiversity conservation at multiple scales: functional sites, landscapes, networks. Bioscience. 2000;50(2):133–46.

[34] Selig ER, Turner WR, Troëng S, Wallace BP, Halpern BS, Kaschner K, et al Global priorities for marine biodiversity conservation. PLoS One. 2014;9(1):e82898 10.1371/journal.pone.0082898 24416151 PMC3885410

[35] Fjeldså J. How broad-scale studies of patterns and processes can serve to guide conservation planning in Africa. Conserv Biol. 2007;21(3):659–67. 10.1111/j.1523-1739.2007.00706.x 17531044

[36] Larsen FW, Rahbek C. Influence of scale on conservation priority setting—a test on African mammals. Biodivers Conserv. 2003;12(3):599–614.

[37] Edgar GJ, Stuart-Smith RD, Willis TJ, Kininmonth S, Baker SC, Banks S, et al Global conservation outcomes depend on marine protected areas with five key features. Nature. 2014;506(7487):216–20. 10.1038/nature13022 24499817

[38] Weeks R, Russ GR, Bucol AA, Alcala AC. Incorporating local tenure in the systematic design of marine protected area networks. Conserv Lett. 2010a; 3(6):445–53.

[39] Roberts CM, McClean CJ, Veron JEN, Hawkins JP, Allen GR, McAllister DE, et al Marine biodiversity hotspots and conservation priorities for tropical reefs. Science. 2002;295(5558):1280–4. 10.1126/science.1067728 11847338

[40] Horigue V, Pressey RL, Mills M, Brotánková J, Cabral R, Andréfouët S. Benefits and challenges of scaling up expansion of marine protected area networks in the Verde Island Passage, Central Philippines. PLoS One. 2015;10(8):e0135789 10.1371/journal.pone.0135789 26288089 PMC4545830

[41] Andréfouët S, Muller-Karger FE, Robinson JA, Kranenburg CJ, Torres-Pulliza D, Spraggins SA, et al., editors. Global assessment of modern coral reef extent and diversity for regional science and management applications: a view from space. Proceedings of 10th International Coral Reef Symposium; 2006.

[42] Soininen J, McDonald R, Hillebrand H. The distance decay of similarity in ecological communities. Ecography. 2007;30(1):3–12.

[43] CNMI Department of Commerce. Northern Marianas Islands: 2010 Census Summary Report 2010 Census of Population and Housing: U.S. Census Bureau; 2010.

[44] Economic Policy Planning and Statistics Office. 1999 Republic of the Marshall Islands Census. Republic of the Marshall Islands; 1999.

[45] Federated States of Micronesia Division of Statistics. 2010 Census of Population and Housing. 2010.

[46] Fiji Bureau of Statistics. 2007 Census of population. 2007.

[47] Office of Planning and Statistics. 2005 Census of Population and Housing of the Republic of Palau. Koror, Palau: Republic of Palau; 2005.

[48] U.S. Census Bureau. 2010 Census for Guam. 2010.

[49] Weeks R, Russ GR, Bucol AA, Alcala AC. Shortcuts for marine conservation planning: the effectiveness of socioeconomic data surrogates. Biol Conserv. 2010b;143(5):1236–44.

[50] Micronesia Challenge. The Micronesia Challenge http://themicronesiachallenge.blogspot.com.au/2012.

[51] Adams VM, Mills M, Jupiter SD, Pressey RL. Improving social acceptability of marine protected area networks: a method for estimating opportunity costs to multiple gear types in both fished and currently unfished areas. Biol Conserv. 2011;144(1):350–61.

[52] Ball IR, Possingham HP, Watts ME. Marxan and relatives: software for spatial conservation prioritisation Spatial conservation prioritisation: quantitative methods and computational tools. Oxford: Oxford University Press; 2009 p. 185–95.

[53] Ardron JA, Possingham HP, Klein. (eds). CJ. Marxan good practices handbook, Version 2 Victoria, BC, Canada: Pacific Marine Analysis and Research Association; 2010 p. 165.

[54] R Core Team. R: a language and environment for statistical computing Vienna, Austria: R Foundation for Statistical Computing; 2014.

[55] Legendre P, Gallagher ED. Ecologically meaningful transformations for ordination of species data. Oecologia. 2001;129(2):271–80. 28547606 10.1007/s004420100716

[56] Oksanen J, Blanchet FG, Kindt R, Legendre P, Minchin PR, O'Hara RB, et al. vegan: Community Ecology Package. R package version 22–1. http://CRAN.R-project.org/package=vegan2015.

[57] Witten DM, Tibshirani R. sparcl: Perform sparse hierarchical clustering and sparse k-means clustering. R package version 103: http://CRAN.R-project.org/package=sparcl; 2013.

[58] Lombard AT, Cowling RM, Pressey RL, Rebelo AG. Effectiveness of land classes as surrogates for species in conservation planning for the Cape Floristic Region. Biol Conserv. 2003;112(1–2):45–62.

[59] Juutinen A, Mäntymaa E, Mönkkönen M, Salmi J. A cost-efficient approach to selecting forest stands for conserving species: a case study from Northern Fennoscandia. Forest Science. 2004;50(4):527–39.

[60] Ban NC, Picard CR, Vincent ACJ. Comparing and integrating community-based and science-based approaches to prioritising marine areas for protection. Conserv Biol. 2009b;23(4):899–910. 19627319 10.1111/j.1523-1739.2009.01185.x

[61] Adams VM, Pressey RL, Naidoo R. Opportunity costs: who really pays for conservation? Biol Conserv. 2010;143(2):439–48.

[62] Delavenne J, Metcalfe K, Smith RJ, Vaz S, Martin CS, Dupuis L, et al Systematic conservation planning in the eastern English Channel: comparing the Marxan and Zonation decision-support tools. ICES J Mar Sci. 2012;69(1):75–83.

[63] Klein CJ, Chan A, Kircher L, Cundiff AJ, Gardner N, Hrovat Y, et al Striking a balance between biodiversity conservation and socioeconomic viability in the design of marine protected areas. Conserv Biol. 2008a;22(3):691–700. 18325043 10.1111/j.1523-1739.2008.00896.x

[64] Mazor T, Giakoumi S, Kark S, Possingham HP. Large-scale conservation planning in a multinational marine environment: cost matters. Ecol Appl. 2014;24(5):1115–30. 25154100 10.1890/13-1249.1

[65] Schröter M, Rusch GM, Barton DN, Blumentrath S, Nordén B. Ecosystem services and opportunity costs shift spatial priorities for conserving forest biodiversity. PLoS One. 2014;9(11):e112557 10.1371/journal.pone.0112557 25393951 PMC4230974

[66] Nhancale BA, Smith RJ. The influence of planning unit characteristics on the efficiency and spatial pattern of systematic conservation planning assessments. Biodivers Conserv. 2011;20(8):1821–35.

[67] Shriner SA, Wilson KR, Flather CH. Reserve networks based on richness hotspots and representation vary with scale. Ecol Appl. 2006;16(5):1660–71. 17069361 10.1890/1051-0761(2006)016[1660:rnborh]2.0.co;2

[68] Warman LD, Sinclair ARE, Scudder GGE, Klinkenberg B, Pressey RL. Sensitivity of systematic reserve selection to decisions about scale, biological data, and targets: case study from Southern British Columbia. Conserv Biol. 2004;18(3):655–66.

[69] Justus J, Fuller T, Sarkar S. Influence of representation targets on the total area of conservation-area networks. Conserv Biol. 2008;22(3):673–82. 10.1111/j.1523-1739.2008.00928.x 18445075

[70] Deas M, Andréfouët S, Léopold M, Guillemot N. Modulation of habitat-based conservation plans by fishery opportunity costs: a New Caledonia case study using fine-scale catch data. PLoS One. 2014;9(5):e97409 10.1371/journal.pone.0097409 24835216 PMC4024024

[71] Pressey RL, Possingham HP, Logan VS, Day JR, Williams PH. Effects of data characteristics on the results of reserve selection algorithms. J Biogeogr. 1999;26(1):179–91.

[72] Giakoumi S, Sini M, Gerovasileiou V, Mazor T, Beher J, Possingham HP, et al Ecoregion-based conservation planning in the Mediterranean: dealing with large-scale heterogeneity. PLoS One. 2013;8(10):e76449 10.1371/journal.pone.0076449 24155901 PMC3796553

[73] Bridge TCL, Grech AM, Pressey RL. Factors influencing incidental representation of previously unknown conservation features in marine protected areas. Conserv Biol. 2016;30(1):154–65. 10.1111/cobi.12557 26040905

[74] Ban NC, Klein CJ. Spatial socioeconomic data as a cost in systematic marine conservation planning. Conserv Lett. 2009;2(5):206–15.

[75] Bode M, Wilson KA, Brooks TM, Turner WR, Mittermeier RA, McBride MF, et al Cost-effective global conservation spending is robust to taxonomic group. Proceedings of the National Academy of Sciences. 2008;105(17):6498–501. 10.1073/pnas.0710705105 PMC2359771 18413614

[76] Kirkpatrick JB, Brown MJ. A comparison of direct and environmental domain approaches to planning reservation of forest higher plant communities and species in Tasmania. Conserv Biol. 1994;8(1):217–24.

[77] Pimm SL, Russell GJ, Gittleman JL, Brooks TM. The future of biodiversity. Science. 1995;269(5222):347–50. 10.1126/science.269.5222.347 17841251

[78] Roberts CM, Hawkins JP. Extinction risk in the sea. Trends Ecol Evol. 1999;14(6):241–6. 10354629 10.1016/s0169-5347(98)01584-5

