# Supplementary figures and images for: Sympathy for the Devil: Detailing the Effects of Planning-Unit Size, Thematic Resolution of Reef Classes, and Socioeconomic Costs on Spatial Priorities for Marine Conservation
Source: PLoS One. 2016 Nov 9;11(11):e0164869. doi: 10.1371/journal.pone.0164869 (PMC5102401; doi:10.1371/journal.pone.0164869)

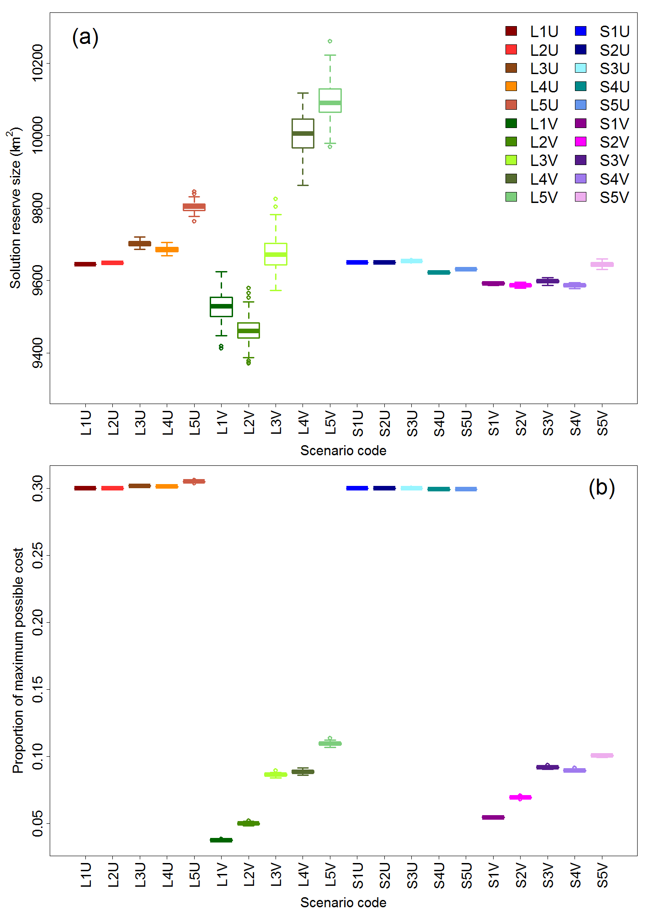

Supplement: S1 Fig — (a) Boxplots of ranges of reserve solution sizes for each scenario based on 100 replicate runs. (b) Boxplots of ranges of total costs (expressed as proportions of maximum possible cost) for each scenario based on 100 replicate runs. Each change in shade of the same colour represents the change in thematic resolution (always presented in order from level 1–5, left to right) for each combination of planning-unit size and cost variability. Colour scheme representing all scenarios remains the same throughout all figures to facilitate interpretation. (TIF) [file pone.0164869.s001.tif]

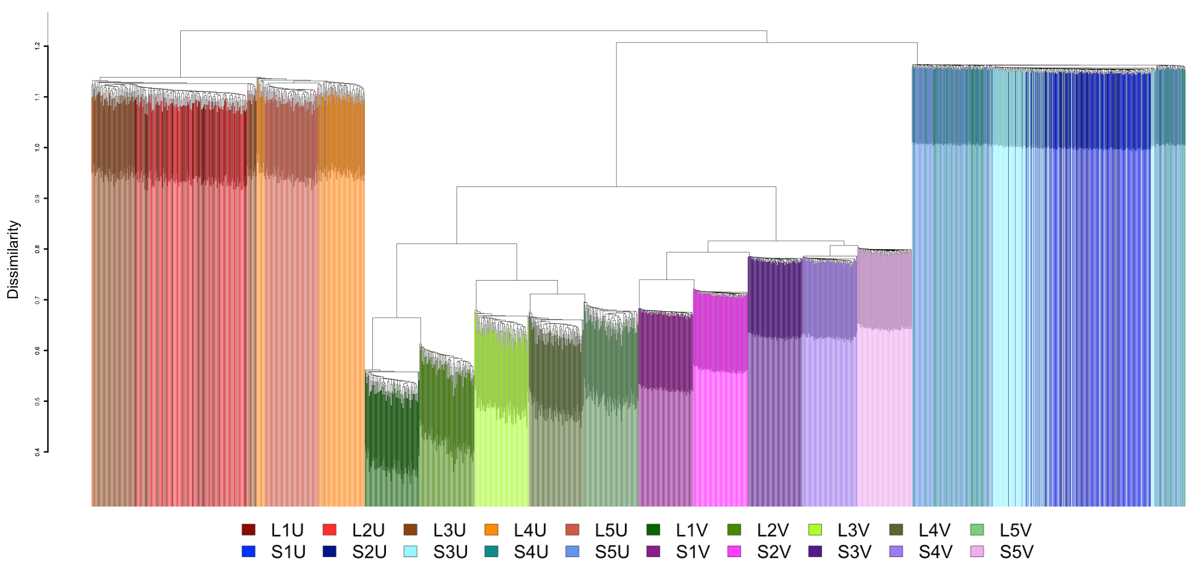

Supplement: S2 Fig — (TIF) [file pone.0164869.s002.tif]

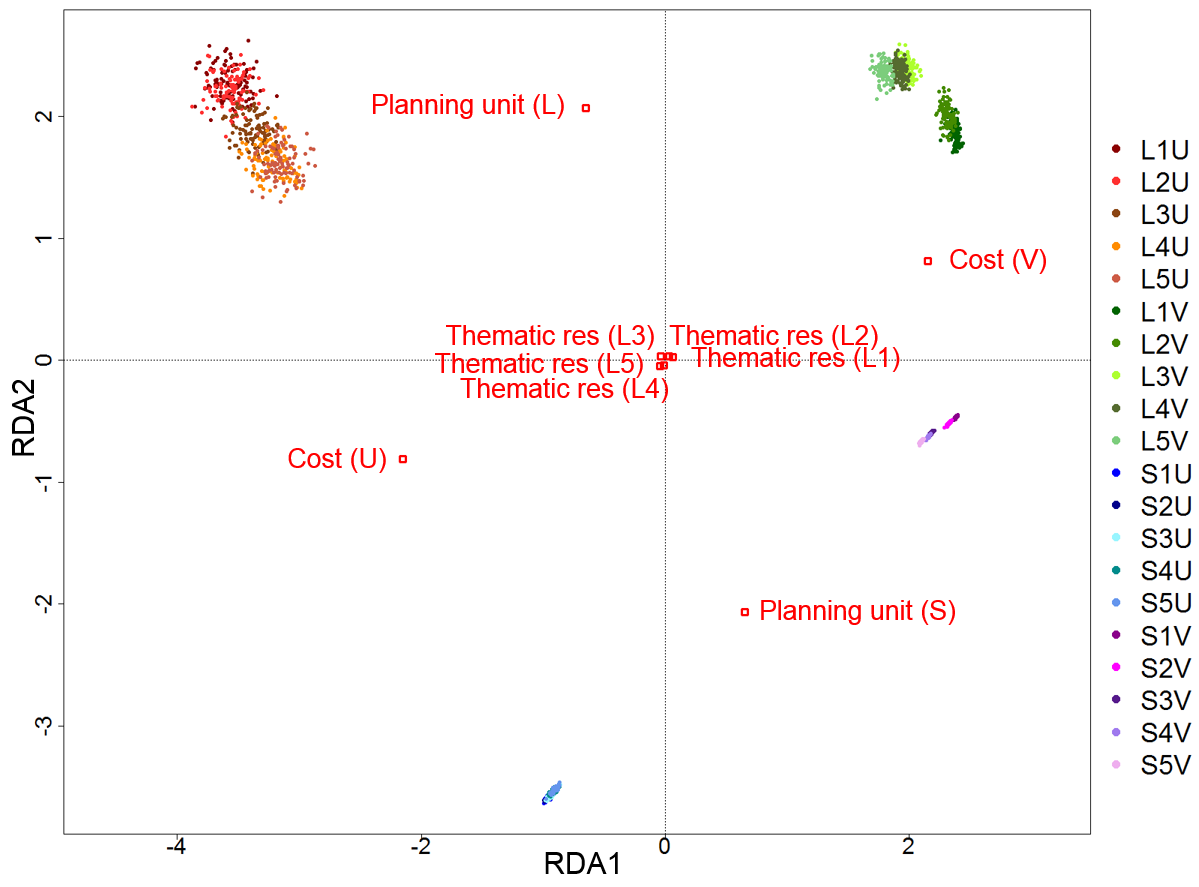

Supplement: S3 Fig — Planning-unit size mainly explains variation along RDA1, while variation along RDA2 is mostly represented by cost variability. Red squares are centroids of the different levels of tested factors, representing the average amount of spatial variance that lines up with the plotted axes. (TIF) [file pone.0164869.s003.tif]

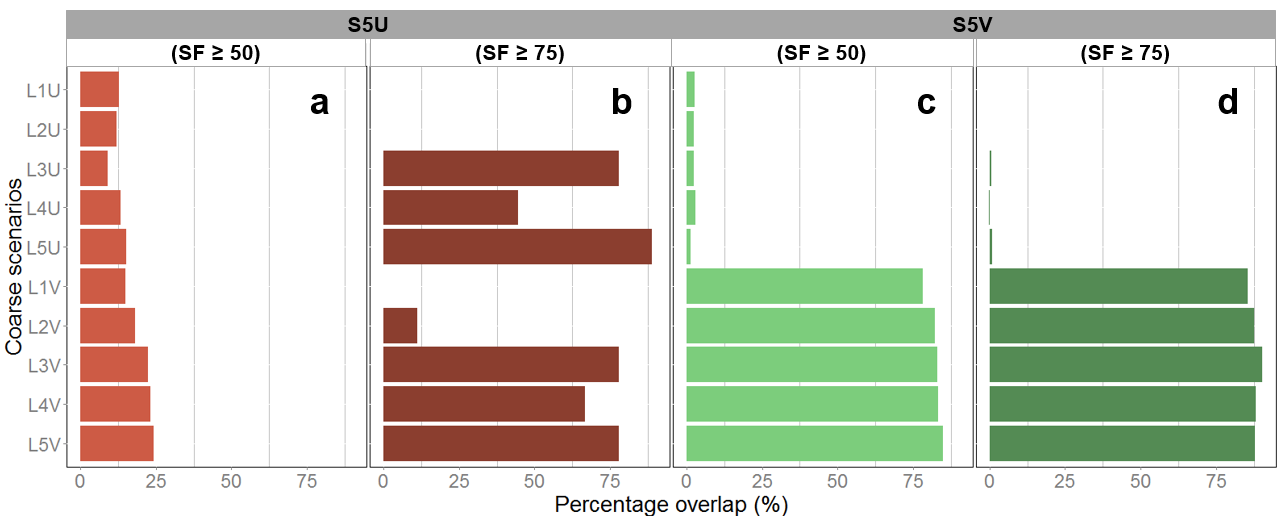

Supplement: S4 Fig — Nestedness of S5U high-priority areas, defined at: (a) selection frequency ≥ 50, and (b) selection frequency ≥ 75. Nestedness of S5V high-priority areas, defined at: (c) selection frequency ≥ 50, and (d) selection frequency ≥ 75. (TIF) [file pone.0164869.s004.tif]

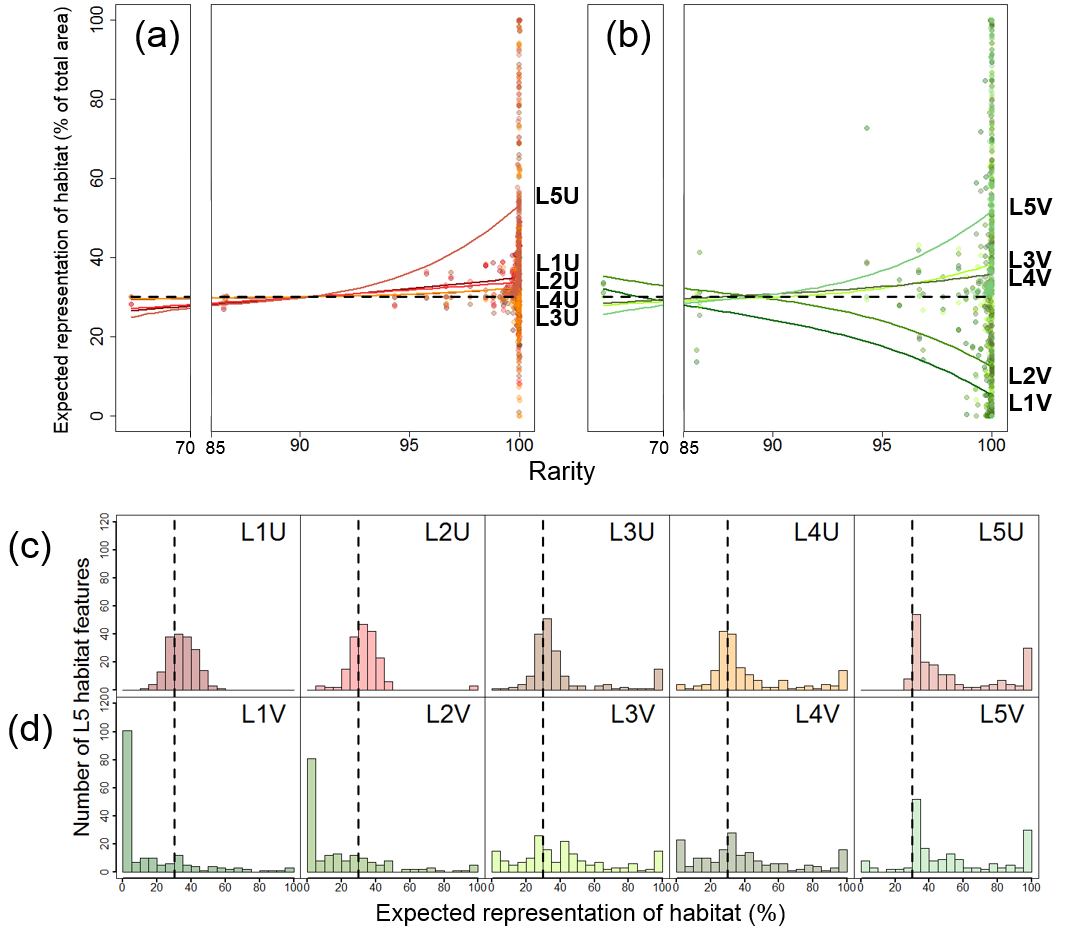

Supplement: S5 Fig — (a-b) Scatter plots showing expected representation of each level 5 reef class (as a percentage of total area of feature occurrence) for each coarse scenario with (a) uniform cost and (b) variable cost, in relation to reef class rarity (transformed to natural log). Due to spread and left-skewness of rarity values, plots are shown with x-axis breaks where no data occur to facilitate interpretation. Local regression (LOESS) curves were fitted for each coarse scenario, indicating non-linear trends in each scatter plot. Dashed horizontal lines represent the 30% objective for level 5 reef classes. (c-d) Histograms showing the distributions of expected representation of level 5 classes for coarse scenarios with (c) uniform cost and (d) variable cost, plotted with 5% bin widths. Dashed vertical lines represent the 30% objective for level 5 classes. (TIF) [file pone.0164869.s005.tif]
