# Supplementary material for: Sympathy for the Devil: Detailing the Effects of Planning-Unit Size, Thematic Resolution of Reef Classes, and Socioeconomic Costs on Spatial Priorities for Marine Conservation
Source: PLoS One. 2016 Nov 9;11(11):e0164869. doi: 10.1371/journal.pone.0164869 (PMC5102401; doi:10.1371/journal.pone.0164869)
Supplement: S1 File — (DOCX) [file pone.0164869.s006.docx]

**S1 File. Additional methods for creating the spatially variable socioeconomic cost layer.**

For both study regions, we derived the variable cost layers by calculating weighted linear distance decays from fisher populations to the furthest reef areas, using fisher densities as a proxy of fishing effort. It is important to note that there are a number of crude assumptions that are necessary when using such a cost layer. First, that distance to fisher populations is a reliable proxy for effort; this assumes that fishers are more likely to travel shorter distances to fish, regardless of their method of transportation. There is also the assumption that fishing pressure decreases linearly across space. Moreover, as this study did not distinguish between subsistence and commercial fishers when calculating fisher densities, the combined costs would likely result in an inequitable impact to the different fishery groups [1]. However, this method of deriving cost is relatively common, particularly in developing countries where explicit socioeconomic data is sparse [2]. Evidence suggests this socioeconomic cost proxy represents cost better than others (e.g. general population numbers or area simply as cost; [3]). Ultimately, the actual cost values and method used to derive these for the spatially variable cost was not so critical for the purposes of this study, since the key aim here was to compare the effects between using spatially uniform and spatially variable costs.

*Spatially variable costs for Fiji*

Total fisher population data were taken from the 2007 census [4]. Census data were collected at the administrative level of Enumeration Areas (EAs), which are designated spatial units across the country for within which census information is collated. A linear distance decay function, weighted to population numbers, was used to simulate the decrease in opportunity cost as distance from fisher populations increased. This was done for two separate distances to represent the two main modes of transport used by fishers: non-motorised transport (e.g. walking, swimming, bilibili/bamboo raft), with costs decreasing to zero at 3 km from shore; and motorised transport (e.g. any boat with an engine) to unlimited distances ([5], online supplementary material). For practical purposes, the “unlimited” distance was set at 87 km, the minimum distance required to ensure that the cost layer covered all mapped coral reef habitats. The only exceptions to this were two small oceanic atolls, in the southern extremity of Fiji’s waters, more than 330 km to the nearest inhabited island. In this case, planning units across these two atolls were designated costs of zero.

Each decay function began at the fisher population number of each censused EA, decreasing linearly to zero for both decay distances (at a resolution of 30m), to be summed together for the final cost layer (Fig 1a). As non-motorised transport is more common compared to motorised transport use amongst fishers, non-motorised and motorised costs were weighted to a ratio of 3:1, respectively. Both cost distances were then rescaled, on a scale of 0–100, to make costs between the different distances and between different datasets relative and allow for direct comparison. Once the weighted costs were summed, specific cost values were attributed to each planning unit based on the mean value of all cost values within the planning unit (Fig 1b).

*Spatially variable costs for Micronesia*

The same procedures were followed for the Micronesia dataset, with two exceptions. As the region includes two U.S. territories and three independent countries, the latest census data was sourced from the respective governments, which were all collected in different years ([6-10]). The same distance decays were used (3km for non-motorised transportation; “unlimited” for motorised transport); however, these distances were weighted differently to Fiji. Due to a more developed socioeconomic context, there are a higher proportion of fishers using motorised boats compared to subsistence canoe or foot-fishers in the region (e.g. [11]). Based on this, the non-motorised and motorised distances were weighted to a ratio of 1:2, respectively.


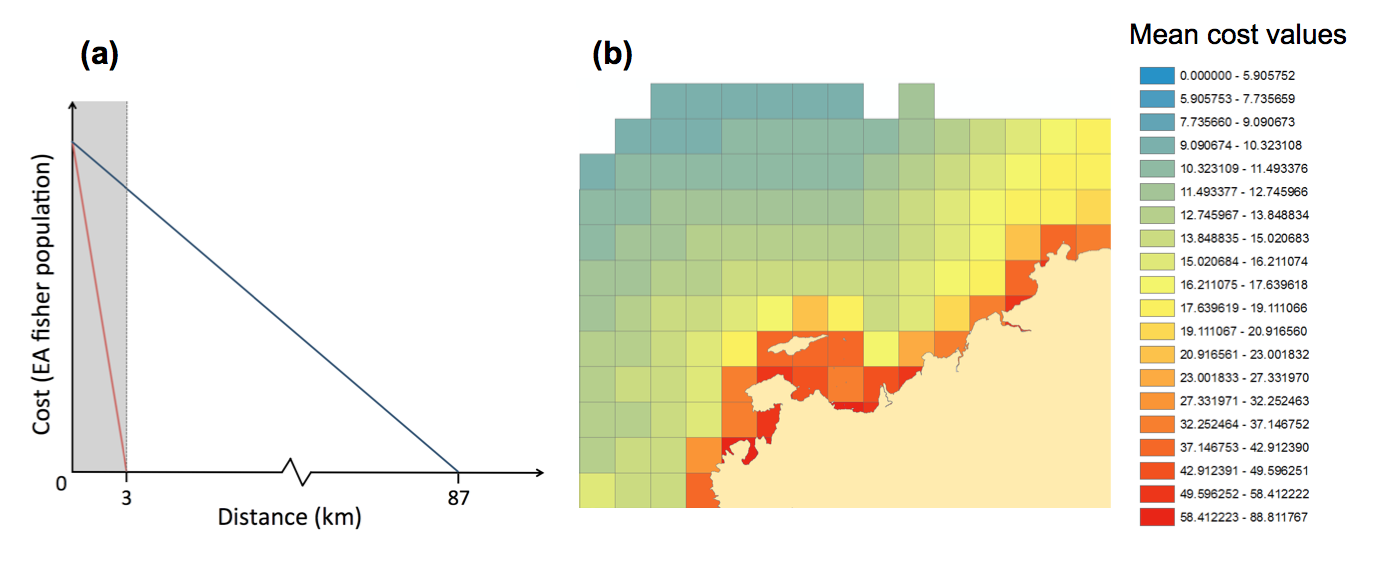


Fig 1. (a) Example of summed cost function (for Fiji). Non-motorised fisher cost (red line, to 3km) was weighted three times more than motorised fisher cost (blue line, to 87km). Where distances overlapped, weighted cost values were summed (grey shaded area). (b) Example section of map of the variable cost layer for large planning units (Fiji dataset).

**References**

1. Ban NC, Klein CJ. Spatial socioeconomic data as a cost in systematic marine conservation planning. Conserv Lett. 2009;2(5):206-15.

2. Ban NC, Hansen GJA, Jones M, Vincent ACJ. Systematic marine conservation planning in data-poor regions: socioeconomic data is essential. Mar Policy. 2009a;33(5):794-800.

3. Weeks R, Russ GR, Bucol AA, Alcala AC. Shortcuts for marine conservation planning: the effectiveness of socioeconomic data surrogates. Biol Conserv. 2010b;143(5):1236-44.

4. Fiji Bureau of Statistics. 2007 Census of population. 2007.

5. Adams VM, Mills M, Jupiter SD, Pressey RL. Improving social acceptability of marine protected area networks: a method for estimating opportunity costs to multiple gear types in both fished and currently unfished areas. Biological Conservation. 2011;144:350-61.

6. CNMI Department of Commerce. Northern Marianas Islands: 2010 Census Summary Report. 2010 Census of Population and Housing: U.S. Census Bureau; 2010.

7. Economic Policy Planning and Statistics Office. 1999 Republic of the Marshall Islands Census. Republic of the Marshall Islands; 1999.

8. Federated States of Micronesia Division of Statistics. 2010 Census of Population and Housing. 2010.

9. Office of Planning and Statistics. 2005 Census of Population and Housing of the Republic of Palau. Koror, Palau: Republic of Palau; 2005.

10. U.S. Census Bureau. 2010 Census for Guam. 2010.

11. Mulyila EJ, Matsuoka T, Anraku K. Sustainability of fishers' communities in tropical island fisheries from the perspectives of resource use and management: a comparative study of Pohnpei (Micronesia), Mafia (Tanzania), and Guimaras (Philippines). Fisheries Science. 2012;78(4):947-64.
